# Hyphal Orientation of *Candida albicans* Is Regulated by a Calcium-Dependent Mechanism

**DOI:** 10.1016/j.cub.2006.12.043

**Published:** 2007-02-20

**Authors:** Alexandra Brand, Scott Shanks, Vanessa M.S. Duncan, Meng Yang, Kevin Mackenzie, Neil A.R. Gow

**Affiliations:** 1 Aberdeen Fungal Group, School of Medical Sciences, Institute of Medical Sciences, University of Aberdeen, Foresterhill, Aberdeen AB25 2ZD, United Kingdom

**Keywords:** MICROBIO, SIGNALING

## Abstract

Eukaryotic cells from fungal hyphae to neurites that grow by polarized extension must coordinate cell growth and cell orientation to enable them to exhibit growth tropisms and to respond to relevant environmental cues. Such cells generally maintain a tip-high Ca^2+^ cytoplasmic gradient, which is correlated with their ability to exhibit polarized tip growth and to respond to growth-directing extracellular signals 1, 2, 3, 4, 5. In yeast and other fungi, the polarisome, exocyst, Arp2/3, and Spitzenkörper protein complexes collectively orchestrate tip growth and cell polarity, but it is not clear whether these molecular complexes also regulate cell orientation or whether they are influenced by cytoplasmic Ca^2+^ gradients. Hyphae of the human pathogenic fungus *Candida albicans* reorient their growth axis in response to underlying surface topography (thigmotropism) [6] and imposed electric fields (galvanotropism) [7]. The establishment and maintenance of directional growth in relation to these environmental cues was Ca^2+^ dependent. Tropisms were attenuated in media containing low Ca^2+^, or calcium-channel blockers, and in mutants where calcium channels or elements of the calcium signaling pathway were deleted. Therefore galvanotropism and thigmotropism may both be mediated by localized Ca^2+^ influx at sites of polarized growth via Ca^2+^ channels that are activated by appropriate environmental signals.

## Results and Discussion

### Calcium Channels in *Candida albicans*

**Figure 1:**
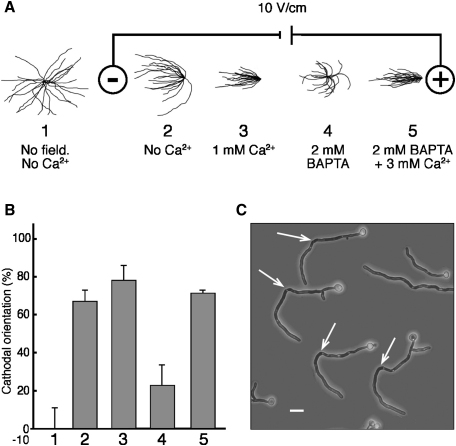
Extracellular [Ca^2+^] Affects Cathodal Emergence of *C. albicans* Hyphae but Not Final Orientation in an Applied Electrical Field (A) Tracings of individual hyphae grown in varying [Ca^2+^] were superimposed at a common point of origin for illustrating the distribution of hyphal orientation under the conditions used. Yeast cells adhered to poly-L-lysine-coated glass slides were grown in Ca^2+^-depleted, hypha-inducing medium for 6 hr and either not exposed to an electrical field (1) or exposed to an electrical field of 10 V/cm (2) supplemented with 1 mM CaSO_4_ (3), 2 mM BAPTA (a Ca^2+^ chelator) (4) or 2 mM BAPTA + 3 mM (excess) CaSO_4_ (5). (B) Germ-tube-emergence angles relative to the cathode for cells in Figure 1A, where 100% cathodal orientation denotes perfect cathodal orientation, −100% denotes anodal orientation, and 0% is obtained for a randomly orientated population. Each error bar shows the SD of the mean values obtained from three independent experiments. (C) The tropic growth of hyphal tips was not affected by extracellular [Ca^2+^]. The final angles of hyphal tips after 6 hr growth in an electrical field were cathodally oriented irrespective of germ-tube-emergence angle. Hyphae reoriented when the field polarity was reversed (arrows), even in low [Ca^2+^] medium. The scale bar represents 10 μM.

Calcium channels have not been identified previously in *C. albicans*, but high-affinity and low-affinity calcium-uptake systems (HACS and LACS) have been described and partially characterized in *S. cerevisiae* 8, 9. We identified and deleted in *C albicans*, homologs of components of these systems—the voltage-gated Cch1p channel [10] and the stretch-activated channel Mid1p [11]. Together, these two channels are thought to form a complex that defines the HACS. We also identified and deleted Fig1p [12]—a component of LACS [13].

*CaCCH1* encodes a putative 2254 amino acid protein with 38.4% identity to its *S. cerevisiae* homolog. The 24 predicted transmembrane (TM) regions in CaCch1p are arranged in four repeated units (I to IV) of six TM domains, as they are in mammalian calcium channels where they tetramerize to form the core α1-subunit of L-type Ca^2+^ channels [14]. The TM regions include segments responsible for voltage-dependency, channel-specificity, and association with organic calcium-channel blockers [15]. The *C. albicans* Cch1p and the human voltage-gated calcium channel CaV1.2 are 62.9% similar and 37.7% identical over a 20 amino acid region in the four Ca^2+^ selective, pore-forming P domains. In the voltage-sensitive S4 domains, 13 of the 23 basic residues in CaV1.2 are identically positioned in CaCch1p. The *CaMID1* gene sequence had 36.9% and 34.4% identity to *ScMID1* and *Schizosaccharomyces pombe yam8*(+), respectively. The 559 amino acid protein it encodes contains four putative TM regions (H1-4), potential *N*-glycosylation sites, a helix-loop-helix domain and 10 conserved cysteines that, in *S. cerevisiae*, form the C1/C2 domains that are essential for activity and localization 11, 16. Unlike in *S. cerevisiae*, the C1/C2 regions in *C. albicans* are located between H3 and the C-terminal H4. CaFig1p shares 48.5% identity with ScFig1p, a putative homolog of mammalian PMP-22/EMP/MP20/Claudins, which are involved in the trafficking and assembly of membrane-associated proteins [17]. Consistent with EMP homology, CaFig1p has four predicted *N*-glycosylation sites in the first of its four TM domains. The role of ScFig1p in *S. cerevisiae* is not well-defined, but it localizes predominantly to the plasma membrane [13] and is required for low-affinity calcium transport and for the calcium-dependent fusion of mating projections [12].

Control strains were created by the generation of conditional mutants expressing a single remaining wild-type gene from the *MRP1* maltose-regulatable promoter (*CCH1* or *MID1*) or by reintegration of the gene at a high-expression locus (*FIG1* or *MID1*) (see the Experimental Procedures in the Supplemental Data available with this article online).

### HACS and LACS Are Expressed during Hyphal Vegetative Growth

In *S. cerevisiae*, HACS is activated by low Ca^2+^ conditions and is regulated by calcineurin, which controls calcium homeostasis and specific stress responses 8, 9, 10, 11, 18. ScFig1p (LACS) activity was only revealed under conditions when HACS was inhibited by rich media. In contrast to HACS, LACS is insensitive to calcineurin and its affinity for Ca^2+^ is 16-fold lower [8]. However, both systems are activated on exposure of cells to α-pheromone, which stimulates the formation of the polarized mating projection 8, 19. Localization of ScMid1p and ScFig1p to the mating projection is dependent on *ScSPA2* and *ScBNI1* 19, 8. In *C. albicans*, CaSpa2p and CaBni1p are components of the hyphal polarisome and Spitzenkörper, respectively, and are required for polarized hyphal extension [20]. Mid1p and Fig1p may therefore be involved in polarized cell growth in both organisms. In *C. albicans*, mRNA encoding HACS and LACS component proteins was detected in both yeast and hyphal growth conditions and in *C. albicans*-infected rabbit kidney (data not shown). Also, both HACS and LACS mutants were affected in the thigmotropic response (see below). Therefore, both HACS and LACS are expressed in *C. albicans* during the normal in vitro and in vivo growth of this fungus.

### The *Cacch1*Δ and *Camid1*Δ Mutants Are Defective in Calcium Accumulation

After prolonged incubation on Ca^2+^-depleted solid minimal medium (>14 days), wild-type *C. albicans* colonies produced aberrant lobed margins that could be alleviated by the addition of 10 mM Ca^2+^ to the medium. Emerging colonies of the *Cacch1*Δ and *Camid1*Δ mutants produced lobed colonies at 2 days (see Supplemental Data). The aberrant morphologies of the colonies of *Cafig1*Δ, *Cacna1*Δ, and *Cacnb1*Δ mutants were partially alleviated on supplementation with exogenous Ca^2+^, supporting the view that these genes are involved in calcium signaling in *C. albicans*. The growth rates of the *Cacch1*Δ, *Camid1*Δ, and *Cacch1*-*mid1*Δ mutants were reduced by 19%, 23%, and 25%, respectively, when they were grown in the yeast form, compared to the control strain (p = <0.037), but the extension rates of hyphae were not affected by the mutations (data not shown). Consistent with the putative roles of CaMid1p and Cch1p as Ca^2+^ channels, Ca^2+^ accumulation in the *Camid1*Δ and *Cacch1*Δ mutants was significantly reduced after 2 hr culture in Ca^2+^-depleted medium supplemented with ^45^Ca^2+^ (p = <0.001; Supplemental Data). Induction of regulatable *CaCCH1* or reintegration of *CaMID1* abrogated this phenotype. The double *Cacch1*Δ*-mid1*Δ mutant had similar Ca^2+^ accumulation and yeast growth rates to the single mutants, consistent with the model that these proteins operate within the same pathway.

Deletion of *CaFIG1* did not affect Ca^2+^ accumulation in low-Ca^2+^ minimal medium. This is consistent with reports that, in *S. cerevisiae*, Cch1-Mid1p are involved in Ca^2+^ homeostasis under low-Ca^2+^ conditions, where Fig1p activity is not detectable [13].

### Calcium Ions and CaCch1p Mediate Cathodal Germ-Tube Emergence

**Figure 2:**
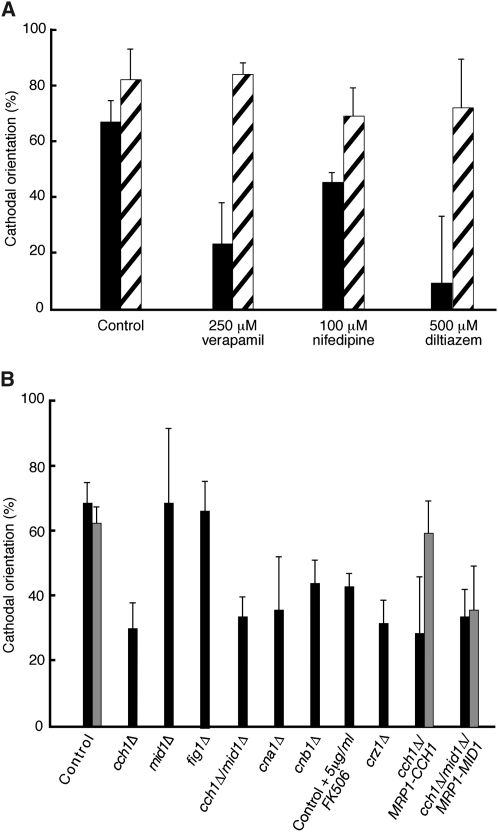
Ca^2+^-Channel Blockers or Deletion of CaCch1p or Either Calcineurin Subunit Attenuates Cathodal Germ-Tube Emergence of *C. albicans* Hyphae (A) Cells were grown in an electric field and the medium supplemented with 250 μM verapamil, 100 μM nifedipine, or 500 μM diltiazem. Emergence angles (black bars) and final angles (hatched bars) were measured in relation to the cathode for >100 hyphae per strain per experiment so that percentage cathodal orientation could be obtained [20]. (B) Mutant and control strains were exposed to an electric field of 10 V/cm for 6 hr in Ca^2+^-depleted medium containing glucose (black bars), maltose (conditional mutants, gray bars), or 5 μg/ml FK506, an inhibitor of calcineurin. Conditional *MRP1p*-regulated mutants were subcultured for 3 days in glucose containing medium prior to assaying. Each error bar shows the SD of the mean values obtained from three independent experiments.

Tropic growth responses to applied external electric fields (galvanotropism) have been observed in migratory and tip-growing cells [21]. Growing *C. albicans* hyphae orient toward the cathode in such fields [7]. To characterize hyphal orientation, we measured the angle at which germ tubes emerged from the mother cell (emergence angle) and the angle of the hyphal tip after 6 hr growth (final angle) relative to the cathode. To investigate the role of calcium ions and channels in galvanotropism, we measured the emergence and final angles of hyphae exposed to electrical fields in media of varying extracellular [Ca^2+^] or in the presence of pharmacological agents that block the activity of L-type voltage-gated cation channels. In *C. albicans*, site selection of germ tubes is not strictly controlled by the Bud proteins that regulate bud-site selection during axial and bipolar budding of *S. cerevisiae* [22]. We observed that, in electric fields, germ tubes were formed almost exclusively on the cathode-facing pole of cells, suggesting that imposed electrical fields can override cortical Bud evagination markers. The percentage cathodal germ-tube emergence was positively correlated with extracellular [Ca^2+^]. Cathodal orientation of wild-type cells was attenuated in Ca^2+^-depleted medium (Figures 1A and 1B) and was further significantly reduced in the presence of calcium-channel blockers (p = <0.05) (Figure 2A). Therefore, Ca^2+^ influx is important for cathodal evagination of the germ tube in *C. albicans*. Localized Ca^2+^ uptake correlates with sites of germination of other cell types, such as the zygotes of the brown alga *Silvetia compressa* [23]. Extracellular [Ca^2+^] did not affect the final angle of hyphae. Even in low Ca^2+^ medium, extending hyphae grew toward the cathode and reoriented their direction of growth when the field polarity was reversed (Figure 1C).

Deletion of *CaCCH1* resulted in a significant reduction in the cathodal orientation of germ-tube emergence (p = <0.001) (Figure 2B). In contrast, emergence angles in the *Camid1*Δ and *Cafig1*Δ mutants were unaffected. Cathodal orientation was restored in the conditional *CaCCH1* strain when *CaCCH1* expression was induced by growth on maltose but not when *CaMID1* was induced in the *cch1*Δ/*mid1*Δ/*MRP1-MID1* strain. We hypothesize that the voltage-gated CaCch1p Ca^2+^ channel is activated by membrane depolarization at the cathodal face of yeast cells and that this results in localized Ca^2+^ uptake and subsequent induction of localized polarized growth.

The effects of extracellular [Ca^2+^] deprivation and *CaCCH1* deletion primarily affected the site of germ-tube emergence. In low [Ca^2+^], in the *Cacch1*Δ mutant and in the presence of L-type Ca^2+^-channel blockers, cathodal germ-tube emergence was approximately half that of the control strain, but after 6 hr exposure to an electric field, this effect was almost lost (Figure 2A). Even hyphae that had emerged from the anodal face of the *Cacch1*Δ mother cell eventually responded to the electric field. This suggested that differences exist between the mechanism that establishes cathodal growth and that which maintains it. Only the former appears to depend on calcium influx and Cch1p.

### CaMid1p, CaCch1p, and CaFig1p Mediate *C. albicans* Thigmotropism

The ability of fungal hyphae to exhibit tropic growth responses in relation to changes in substratum topography is well-known in plant pathogens and has been demonstrated previously for *C. albicans* and certain dermatophytes 24, 25. Some plant pathogenic fungi also use topographical features to trigger formation of the appressorium infection structure (thigmodifferentiation) [26]. We tested whether treatments and mutations that attenuated galvanotropism also influenced thigmotropic orientation in *C. albicans* hyphae.

**Figure 3:**
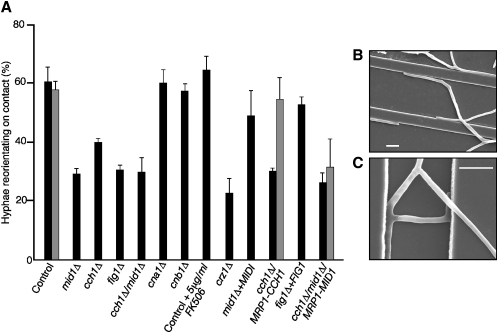
The Thigmotropic Response Is Attenuated in Calcium-Signaling-Pathway Mutants (A) Mutant strains were adhered to quartz slides with ridge height 0.79 μm and pitch 25 μm and grown in 20% (v/v) fetal-calf serum supplemented with 2 (w/v) glucose (black bars), maltose (conditional mutants, gray bars), or a maximal concentration of 10 μg/ml FK506. The number of hypha-ridge interactions resulting in hyphal reorientation was expressed as a percentage of the total number of interactions observed. Each error bar shows the SD of the mean values obtained from three independent experiments. (B and C) In wild-type cells, 60% of interactions between growing tips and the 0.79 μm ridges in the substrate resulted in reorientation of the hyphal growth axis. In mutant strains, approximately 70% of interactions resulted in hyphae maintaining their direction of growth over the ridges. Scale bars represent 10 μm.

**Figure 4:**
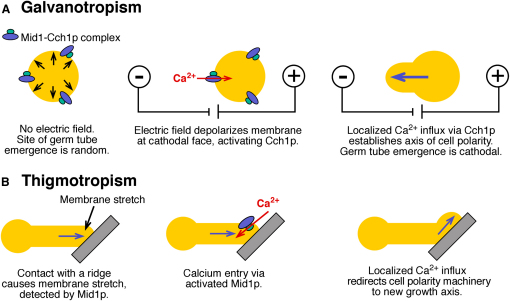
Model for the Role of Ca^2+^ Influx in *C. albicans* Tropic Responses Based on presented data, this model explains how exposure to an extracellular electric field (A) or contact with an obstacle (B) lead to activation of voltage-gated and stretch-activated calcium ion channels, respectively, and hence polarization of the site of germ-tube formation (A) or reorientation of the growth axis (B). The phosphatase, calcineurin, is required for cathodal germ-tube emergence but not for thigmotropism, suggesting that it is involved in interpretation of calcium gradients during the establishment of polarity but not after. The putative Ca^2+^-dependent transcription factor, CaCrz1p, is required for both tropic responses. Its activity may be responsible for the production of proteins involved in sensing or translating environmental signals.

In wild-type cells, 60% of hyphae that contacted a 0.79 μm ridge responded by reorienting their growth axis (Figures 3A and 3B). Deletion of *CaMID1* reduced the number of reorientation events by 50% (p = <0.0001) (Figures 3A and 3C). This is consistent with previous observations that thigmotropism was attenuated by inhibitors of stretch-activated Ca^2+^ channels [6] and supports a model whereby changes in the underlying topography induce stresses in the membrane that are sensed by Mid1p (Figure 4B). Reorientation of the *Cacch1*Δ strain was also significantly reduced (p = <0.001) compared to the control strain (Figure 3). Thigmotropism was attenuated in the regulatable *Cacch1*Δ/*MRP1-CCH1* under repressing conditions and restored to normal under *MRP1p*-inducing conditions. Expression of *CaMID1* in the *Cacch1*Δ/*Camid1*Δ/*MRP1-MID1* conditional mutant did not restore the reorientation response, confirming that CaCch1p is required for thigmotropism. We propose that stretch activation of CaMid1p, and subsequent opening of the CaCch1p channel, results in localized Ca^2+^ influx that exerts an influence on the molecular machinery involved in polarized growth of the hyphal tip. This influence overrides or repositions, or both, the molecular markers that defined the original axis of growth. However, because hyphal reorientation was observed in almost 30% of ridge interactions in the *Camid1*Δ mutant, either CaCch1p is activated independently of CaMid1p or other sensing elements also contribute to the regulation of thigmotropism. Deletion of *CaFIG1* also resulted in attenuation of the reorientation response. The function of Fig1p in *C. albicans* is not known, but in *S. cerevisiae* its deletion resulted in defective cell-cell fusion during mating [12], suggesting it could be involved in the delivery of components to the fusion site. Because its deletion in *C. albicans* reduces hyphal reorientation during contact-sensing, CaFig1p may again be involved in targeted delivery of secretory vesicles to the cell surface. No instances of tip bifurcation were observed when a hyphal tip contacted a ridge, even when the angle of approach was 90° (Figure 3C), suggesting that orientation-determining factors are a nondivisible entity or discrete protein complex.

### Calcium-Signaling Factors Are Required for Both Tropic Responses

Calcium-dependent gene transcription in eukaryotes involves activation of a transcription factor by the phosphatase, calcineurin. Calcineurin regulates fungal morphogenesis [27], calcium flux, and homeostasis. In *C. albicans*, calcineurin acts on the transcription factor, Crz1p [28]. The deletion of *CaCRZ1* affected both cathodal germ-tube emergence (Figure 2B) and thigmotropism (Figure 3), whereas the inhibition of calcineurin with FK506 or deletion of genes encoding either the catalytic CaCna1 or regulatory CaCnb1 calcineurin subunits only affected cathodal emergence (p = 0.006). Calcineurin modulates Cch1p activity [8] and can upregulate expression of *CaCCH1* via Crz1p [28]. Our results suggest that calcineurin is required for the establishment of cathodal cell polarity in an electric field but not for the reorientation of already-polarized hyphal tips during contact-sensing. Thigmotropism was Crz1p dependent but was calcineurin independent. Five genes have been identified previously that are regulated in this manner [28]. Although CaCrz1p influences *CaCCH1* expression, it does not appear to be essential for basal expression or activation of CaMid1p or CaCch1p because the morphology of *Cacrz1*Δ mutant colonies was the same as the wild-type strain, whereas the *Camid1*Δ and *Cacch1*Δ mutants formed aberrant colonies (see Supplemental Data). Other targets of CaCrz1p have been identified in *C. albicans* and are required for resistance to membrane damage and alkaline stress. It is not known whether CaCrz1p-mediated gene expression regulates events that are upstream or downstream of calcium-influx induced tropisms.

## Conclusion

The ability of external cues to influence the orientation of hyphal growth of the human pathogenic fungus, *Candida albicans,* may be relevant to their capacity to infiltrate between human cells during tissue invasion. We have found that reorientation of *C. albicans* hyphae in relation to electrical fields and topographical signals is Ca^2+^ dependent and is mediated by Ca^2+^ channels and a Ca^2+^-dependent transcription factor, CaCrz1p. Calcineurin, the primary regulator of CaCrz1p, was required for galvanotropism but not thigmotropism. We observed that the hyphae of mutants lacking the stretch-activated CaMid1p or voltage-activated CaCch1p proteins grew normally but were attenuated in orientation responses resulting from physical contact or imposed electric fields, respectively. We propose a model whereby localized Ca^2+^-channel activation, caused by localized changes in membrane potential or membrane stretch, results in calcium influx that directs polarized growth (Figure 4). Stretch-activated ion channel activity has been described in patch-clamp analysis of *C. albicans* membranes, but the ion selectivity of these channels is not known [6]. In plant cells, mechanosensory Ca^2+^ channels produce high cytosolic Ca^2+^ and initiation of localized cell-wall expansion at sites of shear stress [29]. Similarly, in mammalian synapses, localized channel activation produces intracellular Ca^2+^ microdomains where the spatial boundary of the domain correlates with the capacity for efficient vesicle exocytosis [30]. Localized Ca^2+^ influx may result in an asymmetry in the tip-high Ca^2+^ gradient in hyphae of *C. albicans* and other fungi. This alters the axis of growth by increasing the rate of vesicle fusion within a local Ca^2+^-high microdomain or by affecting the activity of calcium-binding proteins that are involved in polarized cell extension. Ca^2+^ influx may therefore override existing polarity determinants at the cortex of evaginating mother cells and growing hyphae. Thus, both thigmotropic and galvanotropic responses of *C. albicans* hyphae are dependent on a single Ca^2+^-regulated orientation mechanism.

## Experimental Procedures

For strains used in this study, mutant-strain construction, growth conditions, and determination of Ca^2+^ accumulation, see the Supplemental Experimental Procedures.

### Galvanotropism Assay

Yeast cells were adhered to poly-L-lysine-coated microscope slides and placed on the flat bed of a Biorad midi-sub cell electrophoresis tank [20] and cultured in modified Soll's medium at 37°C ± 1°C for 6 hr at 10 V/cm and a current of 33 ± 2 mA. This field strength could be applied without affecting germ-tube formation or hyphal extension rate, yet it is sufficient for inducing cytoplasmic [Ca^2+^] increases in other cell systems 3, 4. Hyphal orientation at the site of germ-tube emergence and at the germ-tube tip relative to the cathode were measured with Improvision Openlab 2.0 software. The percentage cathodal orientation (*p*) was calculated with *p* = Σ (−sin θ/n) × 100, for n measurements. A minimum of 100 cells was measured in each of three independent experiments for each treatment. Tracings of hyphal growth patterns were generated with Adobe Photoshop.

### Thigmotropism Assay

Yeast cells were adhered to poly-L-lysine-coated quartz slides featuring ridges of 0.79 μm ± 40 nm and a pitch of 25 μm (Kelvin Nanotechnology, Glasgow, UK). This ridge height caused maximal hypha reorientation in a preliminary trial of five heights (data not shown). Slides were placed in 20 ml prewarmed 20% (v/v) newborn-calf serum and 2% (w/v) glucose at 37°C for 6 hr for inducing hyphae. The number of hyphae reorienting on contact with a ridge was expressed as a percentage of the total observed interactions. A minimum of 100 interactions was observed per strain in each experiment, and results were reported as the mean value from three independent experiments ± SD.

## Supplemental Data

Document S1. Experimental Procedures, Two Figures, and One Table
