# Supplementary material for: Hyphal Orientation of Candida albicans Is Regulated by a Calcium-Dependent Mechanism
Source: Curr Biol. 2007 Feb 20;17(4):347–52. doi: 10.1016/j.cub.2006.12.043 (PMC1885950; doi:10.1016/j.cub.2006.12.043)
Supplement: Document S1. Experimental Procedures, Two Figures, and One Table [file mmc1.pdf]

Supplemental Data

Hyphal Orientation of *Candida albicans* Is Regulated by a Calcium-Dependent Mechanism

Alexandra Brand, Scott Shanks,
Vanessa M.S. Duncan, Meng Yang,
Kevin Mackenzie, and Neil A.R. Gow

Supplemental Experimental Procedures

Strains and Growth Media

Strains used in this study are listed in Table S1. Strains were maintained on YPD 1% (w/v) yeast extract, 2% (w/v) mycological peptone, 2% (w/v) glucose, and 2% (w/v) technical agar at room temperature. Media were supplemented with 2% (w/v) glucose or 2% (w/v) maltose for repression and induction of *MRP1*-regulated genes, respectively. For galvanotropism assays, strains were grown in modified Soll's medium (MSM) [S1–S3] containing 5 mM PIPES, where the medium was modified by removal of all chloride salts and by an increase of medium resistivity to 800–1000 Ω cm by 10-fold dilution of the total salt content. From overnight cultures at pH 4.5 and 25°C, hyphae were induced by transfer of inocula into 500 ml MSM [pH 7.5] for 6 hr. The temperature was maintained at 37°C ± 1°C by circulation through a heat exchanger. The medium was supplemented with organic blockers of L-type calcium channels, the calcium chelator, BAPTA (1,2-bis(o-aminophenoxy)ethane- N,N,N′,N′-tetraacetic acid), $CaSO_4$, or 5 μg/ml of the calcineurin inhibitor, FK506, as appropriate. For thigmotropism assays, strains were grown overnight in SC (0.67% [w/v] yeast nitrogen base [YNB] with amino acids, 2% [w/v] glucose) at 30°C, and hyphae were induced by transfer into 20 ml 20% (v/v) newborn-calf serum and 2% (w/v) glucose at 37°C. Concentrations of 1–10 μg/ml FK506 were added for the inhibition of calcineurin in wild-type cells. For growth-rate measurements, Ca^{2+}-accumulation assays, and colony morphology determinations, strains were grown in SD-Ca^{2+} (2% [w/v] glucose, 0.67% [w/v] YNB with ammonium sulfate but without $CaCl_2$). The

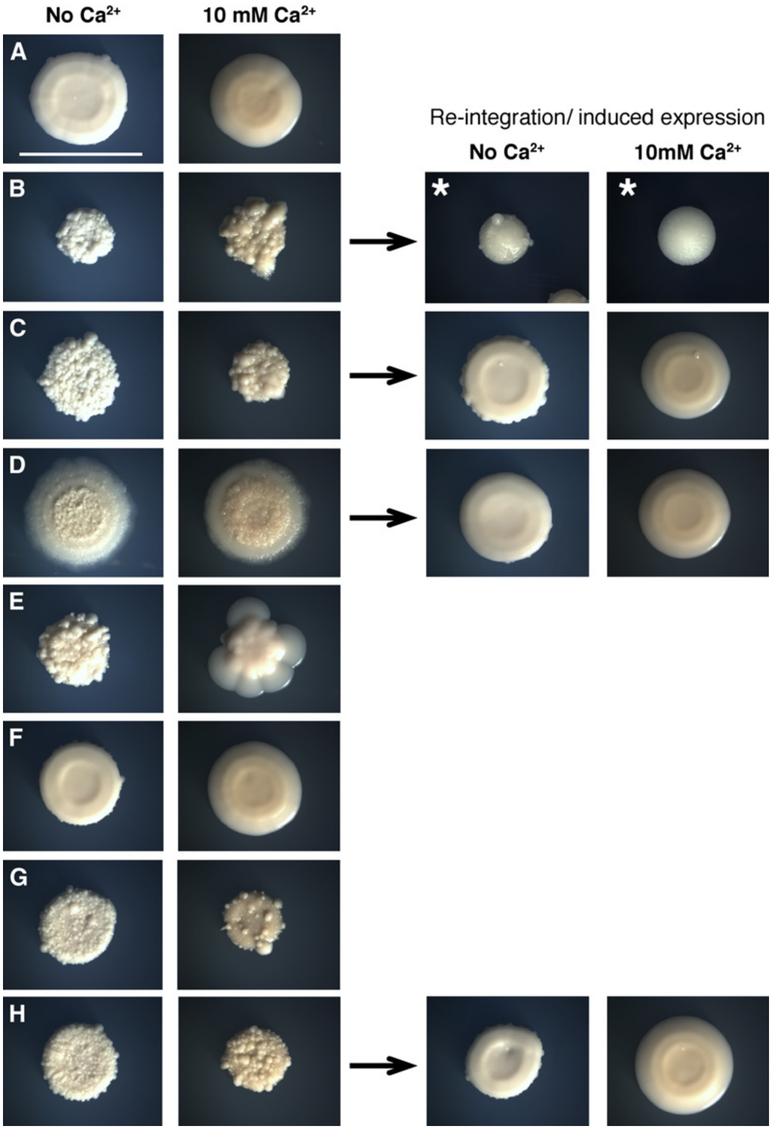

Figure S1. Colony Morphology of *C. albicans* Ca^{2+}-Channel and Ca^{2+}-Signaling Mutants

Yeast cells (5 × 10^4) were spotted on to minimal (YNB) solid medium and grown with or without added Ca^{2+} (10 mM). After 17 days' incubation on Ca^{2+}-depleted solid minimal medium, wild-type *C. albicans* colonies start to produce abberant lobes, which can be alleviated by the addition of 10 mM Ca^{2+} to the medium. In contrast, emerging colonies of the *Cacch1*Δ and *Camid1*Δ mutants were lobed. This morphology was alleviated by re-integration or induced expression of the gene (right) but not by supplementation with Ca^{2+}, suggesting that exogenous Ca^{2+} was not available to the mutant cells. The morphology of *Cafig1*Δ mutant was not lobed but instead invaded the underlying agar. The *Cacrz1*Δ mutant was the same as the wild-type, and the calcineurin mutants (*Cacna1*Δ and *Cacnb1*Δ) were partially lobed. The aberrant morphologies of the *Cafig1*Δ, *Cacna1*Δ, and *Cacnb1*Δ mutants were partially alleviated on supplementation with exogenous Ca^{2+}, supporting the view that these genes are involved in calcium signaling in *C. albicans*. Panels depict the following: (A) control strain, (B) *Cacch1*Δ, (C) *Camid1*Δ, (D) *Cafig1*Δ, (E) *Camid1*Δ*-cch1*Δ, (F) *Cacrz1*Δ, (G) *Cacna1*Δ, and (H) *Cacnb1*Δ. Asterisks denote growth on maltose for inducing *CaCCH1* in conditional strain. The scale bar represents 10 mm.

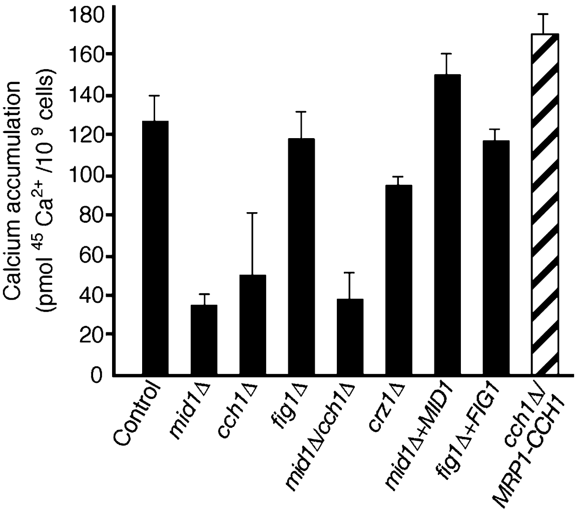

Figure S2. $^{45}Ca^{2+}$ Uptake in the Ca^{2+}-Channel Mutants

Yeast cells were grown in Ca^{2+}-depleted medium prior to incubation with $^{45}Ca^{2+}$ for 2 hr. Hatched bars represent the conditional *CaCCH1* strain normalized to controls grown in inducing conditions (maltose). Each error bar shows the SD of the mean values obtained from three independent experiments.

only source of Ca^{2+} ions in this medium was 0.8 μM Ca^{2+}-pantothenoate, supplementation with Ca^{2+} where stated, or $^{45}Ca^{2+}$ in uptake assays.

Construction of *Cacch1, Camid1,* and *Cafig1* Mutants

Single homologs of *ScCCH1*, *ScMID1*, and *ScFIG1* were identified in the *C. albicans* genome sequence (http://genolist.pasteur.fr/CandidaDB/) as *orf19.3298* (a 6765 bp gene on chromosome 1), *orf19.3212* (a 1677 bp gene on chromosome 5), and *orf19.138* (a 798 bp gene on chromosome 6), respectively. *CaMID1* and *CaCCH1* were disrupted in strain CAI4 in accordance with the standard Ura-blaster method [S4, S5]. For *CCH1*, the downstream region of the gene was amplified with primers 5′-GATCCATACGATGTGACGA-3′ and 5′-GGACATACTATGCTCTCC-3′ and cloned into the Ura-blaster plasmid, pMB-7, to generate plasmid pVC2. The upstream region of the gene was amplified with primers 5′-CTAGACCGAATTCAAGCT-3′ and 5′-TCTCGCACACGTGATAGC-3′ and cloned into pVC2 to generate plasmid pVC3. pVC3 was linearized by digestion with PvuII and transformed into CAI4 to delete 69% of the gene, including all 24 transmembrane domains and the four pore-forming domains. Three rounds of transformation with the Ura-blaster cassette were required for deleting all wild-type copies of *CaCCH1*. Trisomy of genes located on chromosome 1 of *C. albicans* has been reported previously [S6, S7]. For the *mid1*Δ mutant, an upstream sequence of 593 bp (bp 2–594) was amplified by PCR with a SacI site at the 5′ end and a BglII site at the 3′ end, with primers 5′-TGATACCACCTTTT ATTCTACTA-3′ and 5′-CGATTAGTATTTTCCGGTGCTC-3′, and cloned into the Ura-blaster vector, pMB-7, for the creation of plasmid pMB7-CaMID-UP. A downstream sequence of 695 bp, containing the last 371 bp of *MID1*, a 5′ SalI site, and a 3′ HindIII site was amplified with primers 5′-GGTTATGTGCCGTTACTATACCTC-3′ and 5′-GATGATTATTTGTGGGTTTTATAC-3′ and cloned into pMB7-CaMID-UP to generate pSGS2, which was linearized by digestion with HindIII and SacI and used for transforming *C. albicans* strain CAI4. A region comprising 58% of the gene was deleted, including transmembrane domain H3 that is essential for Mid1 function in *S. cerevisiae* [S8]. *CaFIG1* was disrupted by amplification of the *dp1200 URA3* mini-blaster cassette from plasmid pDDB57 [S9] by the use of primers with 19 bp homology to the cassette (underlined) and 111 bp homology to *CaFIG1* (Biomers, Ulm, Germany): 5′-GAG ATATAAAATGAATTTACCATTGAAATTCACCATTGTATTCACAATAA TAATTCAATTCATAACTGCTGCGTTATTAAGTTTCTTATTACTTGGA TGTATAGATACTTCTTTTCCCAGTCACGACGTT-3′ and 5′-GCAGAA ATTTGTACACTAAATTTTTGTATCCACCAGTGGTTGTGGTGGGTGT TGCAGTGATTTTCCATTCAATTTACTCATTCTAATTTCCATCCACCA TGATAAACAAGCTGTGGAATTGTGAGCGGATA-3′. The resulting product was transformed into CAI4, resulting in the deletion of bp 102–702 (75% of the gene).

For avoiding artifacts resulting from ectopic expression of the selectable marker [S10], *URA3* was reintegrated on the StuI-linearized CIp10 plasmid at the *RPS1* locus in the Ura^- derivatives of all mutants [S11]. In control strains, single copies of *FIG1* or *MID1* were introduced at the *RPS1* locus in the null background. For *FIG1*, 734 bp of the promoter region, the 798 bp open reading frame (ORF), and 582 bp of the terminator region were amplified by PCR with primers 5′-ATAACTACGGCTTATTCTGG-3′ and 5′-ACTGCCACTATCATTAA GTC-3′. For *MID1*, a region containing 960 bp of the promoter, the 1641 bp ORF, and 371 bp of the terminator region was amplified with primers 5′-GTTAGAACCTCAACACAATG-3′ and 5′CAAGGCTA

Table S1. *Candida albicans* Strains Used in This Study

Strain	Strain Name	Genotype	Source
CAI4		*ura3Δ-iro1Δ::λimm434/ura3Δ-iro1Δ::λimm434*	[S4, S5]
NGY152	Control strain	As CAI4 but *rps1Δ*::CIp10/*RPS1*	[S10, S11]
NGY171		As CAI4 but *cch1Δ::hisG/cch1Δ::hisG/CCH1*	This study
NGY164		As CAI4 but *cch1Δ::hisG/cch1Δ::hisG/ cch1Δ::hisG*	This study
NGY166	*Cacch1Δ*	As CAI4 but *cch1Δ::hisG/cch1Δ::hisG/ cch1Δ::hisG*, *rps1Δ*::CIp10/*RPS1*	This study
NGY364	*Cacch1Δ/MRP1-CCH1*	As CAI4 but *cch1Δ::hisG/cch1Δ::hisG/MRP1-CCH1*	This study
NGY116		As CAI4 but *mid1Δ::hisG/MID1*	This study
NGY120		As CAI4 but *mid1Δ::hisG/mid1Δ::hisG*	This study
NGY167	*Camid1Δ*	As CAI4 but *mid1Δ::hisG/mid1Δ::hisG*, *rps1Δ*::pCIp10/*RPS1*	This study
NGY468	*Camid1Δ+ MID1*	As CAI4 but *mid1Δ::hisG/mid1Δ::hisG*, *rps1Δ*::CIp10-*MID1/RPS1*	This study
NGY174		As CAI4 but *cch1Δ::hisG/cch1Δ::hisG/cch1Δ::hisG*, *mid1Δ::hisG/MID1*	This study
NGY366	*Cacch1-mid1Δ/MRP1-MID1*	As CAI4 but *cch1Δ::hisG/cch1Δ::hisG/cch1Δ::hisG*, *mid1Δ::hisG/MRP1-MID1*	This study
NGY368	*Cacch1Δ-mid1Δ*	As CAI4 but *cch1Δ::hisG/cch1Δ::hisG/cch1Δ::hisG*, *mid1Δ::hisG/mid1Δ::hisG, rps1Δ*::CIp10/*RPS1*	This study
NGY469		As CAI4 but *fig1Δ::dp1200/fig1Δ::dp1200*	This study
NGY372	*Cafig1Δ*	As CAI4 but *fig1Δ::dp1200/fig1Δ::dp1200*, *rps1Δ*::CIp10/*RPS1*	This study
NGY373	*Cafig1Δ + FIG1*	As CAI4 but *fig1Δ::dp1200/fig1Δ::dp1200*, *rps1Δ*::CIp10-*FIG1/RPS1*	This study
MKY380	*Cacrz1Δ*	As CAI4 but *crz1Δ::hisG/crz1Δ::hisG*, *rps1Δ*::CIp10/*RPS1*	[S15]
DSY2091	*Cacna1Δ*	As CAI4 but *cna1Δ::hisG/cna1Δ::hisG-URA3-hisG*	[S16]
JRB64	*Cacnb1Δ*	As CAI4 but *his1Δ::hisG::HIS1/his1Δ::hisG*, *arg4Δ::hisG/arg4Δ::hisG*, *cnb1Δ::UAU1/cnb1Δ::ARG4*	[S17]
MCC85	*Cacnb1Δ + CNB1*	As CAI4 but *his1Δ::hisG::CNB1-HIS1/his1Δ::hisG*, *arg4Δ::hisG/arg4Δ::hisG*, *cnb1Δ::UAU1/cnb1Δ::ARG4*	[S17]
DAY185		*URA3/ura3Δ-iro1Δ::λimm434*, *HIS1/his1Δ::hisG ARG4/arg4Δ::hisG*	[S18]

TCAGGACGATA-3′. The products were cloned into pGEM-T-Easy (Promega, Southampton, UK) to generate plasmid pYM1 and pAB1, respectively. The inserts were released by digestion with NotI and were subcloned into the CIp10 plasmid to generate plasmids pYM2 and pAB2. These were linearized with StuI and transformed into NGY469 and NGY120, respectively. Expression was confirmed by RT-PCR with primers 5′-GGTTATATGGGTGTATGTTT AT-3′ and 5′-GAAACTAGATTGCTGCATCAT-3′ for *FIG1* and primers 5′-TATATTGTTATGGATTTTGAAGG-3′ and 5′-CTATATGACAACCA TTAAAGTAA-3′ for *MID1*. The *cch1*Δ*/mid1*Δ double null was obtained by disruption of the first *MID1* allele with pSGS2, as described above, in NGY164 to generate strain NGY174. The second *MID1* allele was disrupted by amplification of the *MID1* pSGS2-deleted region from the remaining WT *MID1* allele in NGY174, with primers MIDint-XBA 5′-GCAAATCTAGACTAATCGTACAGCACAATGGACA T-3′ and MIDint-XHO 5′-GCAAACTCGAGCAATTTTTATAACTTTGA GCACAATC-3′. The Xho1-Xba1-digested fragment from this product was used for creating plasmid pDS1197. An internal deletion was made by amplification of pDS1197 with primers MIDint-BGL and MIDint-PST (5′-GCAAAGATCTTTGTTAACCATAAATTGTTGTT-3′ and 5′-GCAAACTGCAGAGTACAAAATATATTGCTTATAT-3′). The product was ligated with a BglII-PstI 3.7 kb fragment from pMB-7 to generate pDS1199. pDS1199 was linearized with ApaI/SacI and transformed into NGY174. CAI4/CIp10 was used as the isogenic control strain in all experiments. As reported for *ScCCH1* [S12], we were unable to isolate full-length *CaCCH1*-containing plasmids from bacteria. We therefore constructed a conditional mutant where the remaining functional allele of *CaCCH1* in NGY171 (*cch1*Δ*/cch1*Δ*/CCH1)* was placed under the control of the maltose/glucose-regulatable *MRP1* promoter [S13]. *CaMID1* was likewise regulated in NGY366 (*cch1*Δ*/cch1*Δ*/cch1*Δ*/mid1/MRP1-MID1)* as a control for *MID1* function in the double null mutant. The *CaCCH1* and *CaMID1* promoters were replaced by the *MRP1* promoter by amplification of the *URA3-MRP1* construct that was previously cloned into pBLUESCRIPT and transformation of the products into the heterozygous mutants NGY171 and NGY174 to generate strains NGY364 and NGY366. For *CaCCH1*, the primers were 5′-GATTCCAAAACTG ATCAAATATTTTAAAGGAAGTTTTCCTAACCTCTGCCAATCTTGCTT TGGATGGTAACACTTTCTAGAAGGACCACCTTTGATTGT-3′ and 5′-ATTTGATTCAGTTGGGGGATTTATTATTATTCCAGGTCTGCCTCGA CTTGAATGGGTATTGCTGTTATCCATGATTTATGATCAAGTTACTTA CAGGTA-3′. For *CaMID1*, the primers were 5′-TAAAACTAAATC AATGTTAGAACCTCAACACAATGATTCAACATCCAATGGTAATGAT AATGGTAGTGGTAATAACCTAGAAGGACCACCTTTGATTGT-3′ and 5′-GGTGAGATTTGTAGATGGATCTAAGTTTTGAAAAAAATCAAATT CAAAAGGTTCACAATATGATGAGTTCATTATTAGAGATCAAGTTAC TTACAGGTA-3′. The *MRP1* promoter is repressed by glucose and induced by maltose in the medium and was selected because changes in carbon source do not impinge on the resistivity of media used in galvanotropism experiments. Conditional *MRP1p*-regulated mutants were subcultured for 3 days in glucose-containing medium prior to assays. *CaCCH1* expression was examined by RT-PCR with primers 5′-CAAGTACATCAACACCAAGT-3′ and 5′-GGGAATTCACC AAATACTTTA-3′. Gene deletion and reintegration was confirmed by Southern analysis (data not shown).

Growth Curves and Ca^{2+} Accumulation

Cells were grown overnight in SD-Ca^{2+} at 30°C with shaking. For yeast-growth assays, cells were washed and diluted to OD_{600} = 0.1 in a 96-well plate (5 wells/strain). Growth was measured spectrophotometrically over 16 hr at 30°C. The length of hyphae grown over 6 hr were measured with Openlab 2.0 software, and the extension rate was calculated. For Ca^{2+} accumulation (adapted from [S14]), cells were washed and diluted to OD_{600} = 1 in 8 ml fresh medium and cell concentrations determined with an Improved Neubauer hemocytometer. Cells were incubated for 2 hr at 30°C, pelleted, and resuspended in 8 ml fresh medium supplemented with 0.6 MBq (megabecquerel) $^{45}Ca^{2+}$ (8 μl of 74 MBq/ml, Amersham Biosciences, Buckinghamshire, UK). The OD_{600} was determined at t = 0 and t = 2 hr, when triplicate samples of 2 ml were filtered with Whatman GC/C filters wetted with medium containing 5 mM calcium and washed with 17 ml of the same. $^{45}Ca^{2+}$ accumulation was determined by scintillation counting and reported as the mean pmol $^{45}Ca^{2+}$ accumulated per 10^9 cells ± SD from three independent experiments.

Scanning Electron Microscopy

Cells were adhered to ridged quartz slides, grown for 6 hr in 20% (v/v) FCS and 2% (w/v) glucose, and fixed with 2.5% glutaraldehyde in 0.1 M phosphate buffer [pH 7.4]. Cells were postfixed with 1% osmium tetroxide, dehydrated in 70%, 90%, 95%, and 100% ethanol, and critical-point-dried in CO_2. The slide was mounted on a stub and sputter-coated with gold. Cells were viewed in a JEOL35CF scanning electron microscope at a voltage of 10 kV.

Supplemental References

S1. Lee, K.L., Buckley, H.R., and Campbell, C.C. (1975). An amino acid liquid synthetic medium for the development of mycelial and yeast forms of *Candida albicans*. Sabouraudia *13*, 148–153.

S2. Buffo, J., Herman, M.A., and Soll, D.R. (1984). A characterization of pH-regulated dimorphism in *Candida albicans*. Mycopathalogia *85*, 21–30.

S3. Crombie, T., Gow, N.A.R., and Gooday, G. (1990). Influence of applied electrical fields on yeast and hyphal growth of *Candida albicans*. J. Gen. Microbiol. *136*, 311–317.

S4. Fonzi, W.A., and Irwin, M.Y. (1993). Isogenic strain construction and gene mapping in *Candida albicans*. Genetics *134*, 717–728.

S5. Garcia, M.G., O'Connor, J.-E., Garcia, L.L., Martinez, S.I., Herrero, E., and del Castillo Agudo, L. (2001). Isolation of a *Candida albicans* gene, tightly linked to *URA3*, coding for a putative transcription factor that suppresses a *Saccharomyces cerevisiae aft1* mutation. Yeast *18*, 301–311.

S6. Chen, X., Magee, B.B., Dawson, D., Magee, P.T., and Kumamoto, C.A. (2004). Chromosome 1 trisomy compromises the virulence of *Candida albicans*. Mol. Microbiol. *51*, 551–565.

S7. Selmecki, A., Bergmann, S., and Berman, J. (2005). Comparative genome hybridization reveals widespread aneuploidy in *Candida albicans* laboratory strains. Mol. Microbiol. *55*, 1553–1565.

S8. Tada, T., Ohmori, M., and Iida, H. (2003). Molecular dissection of the hydrophobic segments H3 and H4 of the yeast Ca^{2+} channel component Mid1. J. Biol. Chem. *278*, 9647–9654.

S9. Wilson, R.B., Davis, D., Enloe, B.M., and Mitchell, A.P. (2000). A recyclable *Candida albicans URA3* cassette for PCR product-directed gene disruptions. Yeast *16*, 65–70.

S10. Brand, A., MacCallum, D.M., Brown, A.J.P., Gow, N.A.R., and Odds, F.C. (2004). Ectopic expression of *URA3* can influence the virulence phenotypes and proteome of *Candida albicans* but can be overcome by targeted reintegration of *URA3* at the *RPS10* locus. Eukaryot. Cell *3*, 900–909.

S11. Murad, A.M., Lee, P.R., Broadbent, I.D., Barelle, C.J., and Brown, A.J. (2000). CIp10, an efficient and convenient integrating vector for *Candida albicans*. Yeast *16*, 325–327.

S12. Paidhungat, M., and Garrett, S. (1997). A homolog of mammalian, voltage-gated calcium channels mediates yeast pheromone-stimulated Ca^{2+} uptake and exacerbates the cdc1(Ts) growth defect. Mol. Cell. Biol. *17*, 6339–6347.

S13. Munro, C.A., Winter, K., Buchan, A., Henry, K., Becker, J.M., Brown, A.J.P., Bulawa, C.E., and Gow, N.A. (2001). Chs1 of *Candida albicans* is an essential chitin synthase required for synthesis of the septum and for cell integrity. Mol. Microbiol. *39*, 1414–1426.

S14. Ozeki-Miyawaki, C., Moriya, Y., Tatsumi, H., Iida, H., and Sokabe, M. (2005). Identification of functional domains of Mid1, a stretch-activated channel component, necessary for localization to the plasma membrane and Ca^{2+} permeation. Exp. Cell Res. *311*, 84–95.

S15. Karababa, M., Valentino, E., Pardini, G., Coste, A.T., Bille, J., and Sanglard, D. (2006). *CRZ1*, a target of the calcineurin pathway in *Candida albicans*. Mol. Microbiol. *59*, 1429–1451.

S16. Sanglard, D., Ischer, F., Marchetti, O., Entenza, J., and Bille, J. (2003). Calcineurin A of *Candida albicans*: Involvement in antifungal tolerance, cell morphogenesis and virulence. Mol. Microbiol. *48*, 959–976.

S17. Cruz, M.C., Goldstein, A.L., Blankenship, J.R., del Poeta, M., Davis, D., Cardenas, M.E., Perfect, J.R., McCusker, J.H., and

Heitman, J. (2002). Calcineurin is essential for survival during membrane stress in *Candida albicans*. EMBO J. *21*, 546–559.

S18. Davis, D., Edwards, J.E., Jr., Mitchell, A.P., and Ibrahim, A.S. (2000). *Candida albicans* RIM101 pH response pathway is required for host-pathogen interactions. Infect. Immun. *68*, 5953–5959.
